# Clinical Characteristics and Outcomes of COVID-19 Acute Respiratory Distress Syndrome (ARDS) Survivors in Early Pandemic: A Single Healthcare System Retrospective Study

**DOI:** 10.7759/cureus.26801

**Published:** 2022-07-13

**Authors:** Cyrus A Vahdatpour, Sheila De Young, Johnny F Jaber, Joy Ayyoub, Thomas Sommers, Christopher Wen, Tristan Lim, Olutosin Owoyemi, Kathleen Davin, Paul Kinniry, Onyeka Nwankwo, Adam Austin

**Affiliations:** 1 Pulmonary and Critical Care Medicine, University of Florida, Gainesville, USA; 2 Medicine, Pennsylvania Hospital, Philadelphia, USA; 3 Pulmonary and Critical Care Medicine, University of Florida College of Medicine, Gainesville, USA; 4 Pulmonary and Critical Care Medicine, Temple University Hospital, Philadelphia, USA; 5 Medical Education, University of Pennsylvania Perelman School of Medicine, Philadelphia, USA; 6 Pulmonary and Critical Care Medicine, Pennsylvania Hospital, Philadelphia, USA; 7 Infectious Diseases, Pennsylvania Hospital, Philadelphia, USA

**Keywords:** covid-19 respiratory failure, critical care outcomes, invasive mechanical ventilation, covid 19, acute respiratory distress syndrome [ards]

## Abstract

Introduction: Acute respiratory distress syndrome (ARDS) management in the intensive care unit (ICU) has attracted strong interest since the start of the COVID-19 pandemic. Our retrospective study aims to describe the outcomes and predictors of mortality of ARDS associated with COVID-19 within one university-based healthcare system.

Methods: We identified 165 patients within our healthcare system during the months of April 2020 through July 2020, who were admitted to our medical ICUs and eligible for our study. Baseline patient characteristics, ICU and hospital course information, ICU interventions, ventilator settings, and hospital complications were collected and analyzed using descriptive statistical techniques.

Results: Our cohort had an average age of 64. No significant difference in mortality was identified with male vs. female gender or BMI. Most of the patient cohort was identified as black (68.2%). The overall mortality of our cohort was 38.2%. Hyperlipidemia, coronary artery disease, and chronic obstructive pulmonary disease were all associated with higher mortality. There was a significant difference in mortality between those with higher observed ventilator plateau pressures at 24 hours and higher driving pressures at 24 hours.

Conclusion: COVID-19-associated ARDS is associated with significant mortality. Physicians should be aware of pre-existing conditions potentially related to worse outcomes so that they receive an appropriate level of care in a timely manner. Ventilator management should focus on maintaining low intra-thoracic pressure changes. Prospective studies are needed to guide COVID-19-associated ARDS management.

## Introduction

Since the introduction of coronavirus disease 2019 (COVID-19) in late 2019, there has been a race to identify preventative measures, therapeutics, and predictors of populations at most risk for severe disease. This unprecedented rise in research has guided the introduction of novel and repurposed therapeutics in managing patients with COVID-19 at nearly every stage of the disease. Early studies attempted to disseminate clinical recommendations to standardize care, while also predicting potential adverse outcomes in patients with severe diseases [1-4]. The landscape of therapeutics has evolved dramatically since the start of the pandemic, posing a challenge in comparing studies around the world and even across health centers.

Approximately 5%-10% of cases require intensive care with severe symptoms that result in respiratory failure and multiorgan failure [4-5]. Mortality in critically-ill patients with COVID-19 has been reported anywhere between 27.2% and 48.8%[2, 5], and respiratory complications are the most common cause of mortality [6]. Despite efforts to develop targeted pharmaceutical therapy to treat this disease, there remains limited data to support optimal settings for mechanical ventilation and for management of adjuvant therapies in COVID-19-associated acute respiratory distress syndrome (ARDS).

The clinical features of patients with COVID-19-associated ARDS, ventilator management, ICU complications, and outcomes have been investigated in a few observational studies. In this retrospective observational study, our primary objective is to describe the clinical outcomes and predictors of mortality of patients with COVID-19-associated ARDS within one healthcare system. We attempt to compare the survivors and non-survivors of COVID-19-associated ARDS in our hospital system during the early phase of the pandemic.

## Materials and methods

The Institutional Review Board (IRB) at the University of Pennsylvania approved this study (IRB# 842904). We performed a retrospective analysis from April 1, 2020, through July 31, 2020. Study subjects were admitted to the medical ICU from three different hospitals within the University of Pennsylvania Health System and identified based on the international classification of disease (ICD) coding. We collected patient data from electronic medical records (EMRs). 

Patients were included in the analysis if there was an admission to an ICU with COVID-19-associated ARDS. The diagnosis of COVID-19 was established with a positive severe acute respiratory syndrome coronavirus 2 (SARS‑CoV-2) by polymerase chain reaction testing, and an ARDS diagnosis according to the Berlin Criteria [7]. The study population had to require either a high flow nasal cannula, non-invasive positive pressure ventilation, or mechanical ventilation for at least 24 h. Patients were excluded if they were on chronic steroids or had a tracheostomy prior to admission. For those with multiple ICU admissions related to their COVID-19 infection, only their initial presentation was used in data extraction and analysis.

Baseline clinical characteristics including age, gender, body mass index (BMI), and medical comorbidities were collected. The type of supportive oxygen therapy, number of supportive oxygen therapy days, type of steroid therapy received, as well as peak and 24-h ventilatory settings were also identified. The peak ventilatory setting was defined as the highest documented setting on EMR respiratory flow sheet any time after the first 24-h interval. Clinical variables were defined as shown in the Appendices.

Statistical analyses

We summarized the distribution of patient characteristics and outcomes as percentages for categorical variables and means with standard deviations for all continuous variables. The Shapiro-Wilk test was used to determine distribution normality. Pending the outcome of this, continuous variables were compared either using an independent sample t-test or a two-sample Wilcoxon rank-sum test. All dichotomous variables were analyzed in a univariate fashion using either Pearson’s Chi-square or Fisher’s exact tests. Significance was determined at a threshold of ɑ = 0.05. All statistical analyses were performed using JMP Pro for Windows, version 15.1 (SAS Institute Inc., Cary NC, 1989-2020).

## Results

One hundred and sixty-five ICU patients with COVID-19-associated ARDS were included in our study population. Of the 393 patients identified, 228 patients were excluded (Figure 1). Baseline clinical characteristics and comorbidities of our study population are outlined in Table 1. Survivors at discharge were younger than those who died during admission (59.8 vs. 69.2, p<0.001). Patients primarily identified were black (68.2%). Most patients had a prior or active history of tobacco use (57.4%). Non-survivors were more likely to have a prior medical history of hyperlipidemia, coronary artery disease, and chronic obstructive pulmonary disease (Table 1).

**Table 1:** Demographics of patients with COVID-19 and ARDS. COVID-19, coronavirus disease 2019; ARDS, acute respiratory distress syndrome; COPD, chronic obstructive pulmonary disease; CVA, cerebral vascular accident; ILD, interstitial lung disease; OHS, obesity hyperventilation syndrome; OSA, obstructive sleep apnea; SD, standard deviation; TIA, transient ischemic attack; BMI, body mass index *Indicates a statistically significant result at ⍺ = 0.05

Characteristic	Total population (n = 165)	Survivors (n = 85)	Non-survivors (n = 63)	Significance (p-value)	
Age (Mean, SD)	64.3 (14.5)	59.8 (14.2)	69.2 (13.6)	<0.001*	
Male gender (n, %)	73 (49.3)	43 (58.9)	30 (41.1)	0.721	
BMI (Mean, SD)	32.6 (10.2)	32.8 (8.4)	33.0 (12.1)	0.906	
Race					
Caucasian (n, %)	25 (15.2)	12 (52.2)	11 (47.8)	0.579	
Hispanic (n, %)	10 (6.1)	6 (60.0)	4 (40.0)	0.860	
Black (n, %)	109 (66.1)	60 (59.4)	41 (40.6)	0.477	
Asian (n, %)	8 (4.9)	3 (42.9)	4 (57.1)	0.424	
History of tobacco use (n, %)	85 (57.4)	44 (51.8)	41 (48.2)	0.105	
Prior medical history					
Hypertension (n, %)	120 (72.7)	62 (55.8)	49 (44.1)	0502	
Diabetes mellitus (n, %)	83 (56.4)	51 (59.3)	35 (40.7)	0.588	
Hyperlipidemia (n, %)	67 (40.6)	29 (46.0)	34 (54.0)	0.016*	
Coronary artery disease (n, %)	25 (15.2)	8 (34.8)	15 (65.2)	0.017*	
Pulmonary hypertension (n, %)	5 (3.0)	3 (60.0)	2 (40.0)	0.906	
Atrial Flutter/Fibrillation (n, %)	17 (10.3)	9 (56.3)	7 (43.8)	0.919	
Venous thromboembolism (n, %)	23 (13.9)	11 (55.0)	9 (45.0)	0.813	
Chronic kidney disease (n, %)	40 (24.2)	17 (47.2)	19 (52.8)	0.154	
COPD (n, %)	35 (21.2)	11 (36.7)	19 (63.3)	0.010*	
ILD (n, %)	4 (2.4)	2 (66.7)	1 (33.3)	0.744	
OSA/OHS (n, %)	37 (22.4)	21 (60.0)	14 (40.0)	0.725	
CVA/TIA (n, %)	29 (17.6)	13 (50.0)	13 50.0)	0.399	
Malignancy (n, %)	27 (16.4)	14 (53.9)	12 (46.2)	0.684	

Overall, those on fewer days of overall of supportive oxygen therapy had a higher mortality rate (29.9 vs. 19.4 days, p=0.001) (Table 2). Survivors had higher numbers of days on nasal cannula (7.4 vs. 1.9 days, p<0.001). Survivors were more like to receive a tracheostomy (79.1% vs. 20.9%, p=0.001) or have extubation (94.2% vs. 5.8%, p<0.001). Of those who received steroids, those on hydrocortisone had higher mortality (40.9% vs. 59.1%, p=0.008). Those on methylprednisolone had lower mortality (66.7% vs. 33.3%, p=0.013).

**Table 2:** Supplemental oxygenation, steroid use, and patient outcomes in COVID-19 ARDS. BIPAP, bilevel positive airway pressure; HFNC, high flow via nasal cannula; LTAC, long term acute care hospital; NC, nasal cannula; SD, standard deviation; ARDS, acute respiratory distress syndrome *Indicates a statistically significant result at ⍺ = 0.05

Characteristic	Total population (n = 165)	Survivors (n = 85)	Non-survivors (n = 63)	Significance (p-value)	
Days on Supportive Oxygen Therapy (Mean, SD)	25.5 (19.3)	29.9 (20.1)	19.4 (16.0)	0.001*	
Days on NC (Mean, SD)	5.0 (6.7)	7.4 (7.9)	1.9 (2.8)	<0.001*	
Days on HFNC (Mean, SD)	2.2 (3.4)	2.5 (3.5)	1.9 (3.3)	0.309	
Days on BIPAP (Mean, SD)	1.2 (3.8)	1.6 (4.8)	0.7 (2.2)	0.168	
Days on mechanical ventilation (Mean, SD)	17.8 (16.1)	18.9 (16.8)	15.6 (14.5)	0.180	
Patients on Steroid Therapy					
Hydrocortisone	44 (29.7)	18 (40.9)	26 (59.1)	0.008*	
Methylprednisolone	81 (54.7)	54 (66.7)	27 (33.3)	0.013*	
Dexamethasone	31 (21.0)	20 (64.5)	11 (35.5)	0.370	
None	32 (21.6)	15 (46.9)	17 (53.1)	0.173	
Outcomes					
Tracheostomy (n, %)	44 (29.1)	34 (79.1)	9 (20.9)	0.001*	
Extubation (n, %)	71 (49.3)	65 (94.2)	4 (5.8)	<0.001*	
Discharge home (n, %)	66 (40.0)	-	-	-	
Discharge to LTAC (n, %)	20 (12.1)	-	-	-	

Table 3 summarizes patients who were mechanically ventilated with both COVID-19 and ARDS. Some 27.1% were diagnosed with mild ARDS, 40.5% with moderate ARDS, and 9.5% with severe ARDS at 24 h, which did not have any significant bearing on mortality. At 24 h, those who were mechanically ventilated and died had on average higher FiO2 (0.48 vs. 0.56, p=0.008), higher plateau pressures (23.2 vs. 25.6, p=0.023), and higher driving pressures (11.8 vs. 13.5, p=0.048). At their peak settings, those who died had significantly higher FiO2 (0.71 vs. 0.83, p=0.006), higher average airway pressures (20.4 vs. 24.2, p=0.004), higher positive end expiratory pressure (PEEP) (12.4 vs. 14.4, p=0.012), higher peak airway pressures (40.6 vs. 44.3, p=0.029), and higher plateau pressures (29.0 vs. 32.6, p=0.009). Those who survived had a higher average nadir PaO2/FiO2 ratio (116.4 vs. 89.9, p=0.005).

**Table 3:** Ventilator settings at 24 h and at peak in patients with COVID-19 ARDS. ARDS, acute respiratory distress syndrome; MAP, mean arterial pressure; PEEP, positive end expiratory pressure; P:F, pressure of arterial oxygen to fraction of inspired oxygen; RR, respiratory rate; TV, tidal volume *Indicates a statistically significant result at ⍺ = 0.05

Characteristic	Total Population (n = 165)	Survivors (n = 85)	Non-Survivors (n = 63)	Significance (p-value)
ARDS severity (24 h)				
Mild (n, %)	41 (27.1)	27 (65.9)	14 (34.2)	0.200
Moderate (n, %)	60 (40.5)	33 (55.0)	27 (45.0)	0.621
Severe (n, %)	14 (9.5)	6 (42.9)	8 (57.1)	0.246
Ventilator settings (24 h)				
RR (Mean, SD)	26.5 (6.3)	26.3 (6.6)	26.9 (5.9)	0.591
TV (Mean, SD)	388.3 (63.5)	389.4 (65.3)	384.5 (62.5)	0.670
FiO2 (Mean, SD)	0.52 (0.17)	0.48 (0.14)	0.56 (0.18)	0.008*
PEEP (Mean, SD)	11.5 (3.8)	11.3 (3.9)	11.9 (3.7)	0.400
Peak airway pressure (Mean, SD)	28.5 (6.9)	28.3 (6.1)	28.9 (8.0)	0.667
Average airway pressure (Mean, SD)	16.6 (5.0)	16.2 (4.8)	17.1 (5.4)	0.359
Plateau pressure (Mean, SD)	24.2 (5.3)	23.2 (4.3)	25.6 (6.3)	0.023*
Driving pressure (Mean, SD)	12.5 (4.2)	11.8 (2.7)	13.5 (5.6)	0.048*
Ventilator settings (Peak)				
RR (Mean, SD)	31.5 (6.5)	30.9 (6.9)	32.6 (6.0)	0.144
TV (Mean, SD)	434.2 (86.6)	435.7 (80.5)	430.6 (95.2)	0.744
FiO2 (Mean, SD)	0.75 (0.26)	0.71 (0.26)	0.83 (0.24)	0.006*
PEEP (Mean, SD)	13.3 (4.4)	12.4 (3.8)	14.4 (4.9)	0.012*
MAP (Mean, SD)	22.0 (7.3)	20.4 (5.7)	24.2 (8.4)	0.004*
Peak airway pressure (Mean, SD)	42.4 (11.8)	40.6 (11.6)	44.3 (10.6)	0.029*
Plateau pressure (Mean, SD)	30.6 (7.6)	29.0 (6.5)	32.6 (8.6)	0.009*
Driving pressure (Mean, SD)	17.2 (5.7)	16.5 (5.2)	17.9 (6.2)	0.168
Lowest P/F ratio	104.5 (56.7)	116.4 (62.0)	89.9 (45.6)	0.005*

Patients who received steroids were compared with those who did not in Table 4. Patients identified as black were more likely to receive steroids (85.3% vs. 14.7%, p=0.033). The duration of high flow nasal cannula (HFNC) and bilevel positive airway pressure (BIPAP) therapy was longer in those who received steroids in comparison with those who did not (2.5 vs. 0.9 days, p=0.001; 1.4 vs. 0.2, p=0.007, respectively). Patients on steroids were also more likely to experience hyperglycemia and delirium (88.4% vs. 11.6%, p=0.009; 69.2% vs. 30.8%, p=0.012, respectively).

**Table 4:** Comparison of patients who received steroids vs. no steroids. ARDS, acute respiratory distress syndrome; BIPAP, bilevel positive airway pressure; BMI, body mass index; COPD, chronic obstructive pulmonary disease; CVA, cerebral vascular accident; HFNC, high flow via nasal cannula; ILD, interstitial lung disease; LTAC, long term acute care hospital; MAP, mean arterial pressure; NC, nasal cannula; OHS, obesity hypoventilation syndrome; OSA, obstructive sleep apnea; PEEP, positive end expiratory pressure; P:F, pressure of arterial oxygen to fraction of inspired oxygen; RR, respiratory rate; SD, standard deviation; TIA, transient ischemic attack; TV, tidal volume *Indicates a statistically significant result at ⍺ = 0.05

Characteristic	Total population (n = 165)	Steroids (n = 133)	No steroids (n = 32)	Significance (p-value)
Age (Mean, SD)	64.3 (14.5)	63.5 (14.2)	67.1 (15.4)	0.237
Male gender (n, %)	81 (50.9)	67 (82.7)	14 (17.3)	0.362
BMI (Mean, SD)	32.6 (10.2)	32.8 (9.8)	31.9 (11.8)	0.698
Race				
Caucasian (n, %)	25 (15.2)	17 (68.0)	8 (32.0)	0.084
Hispanic (n, %)	10 (6.1)	6 (60.0)	4 (40.0)	0.089
Black (n, %)	109 (66.1)	93 (85.3)	16 (14.7)	0.033*
Asian (n, %)	8 (4.9)	7 (87.5)	1 (12.5)	0.613
History of tobacco use (n, %)	99 (60.0)	84 (84.9)	15 (15.2)	0.091
Prior medical history				
Hypertension (n, %)	120 (72.7)	96 (80.0)	24 (20.0)	0.748
Diabetes mellitus (n, %)	83 (56.4)	77 (82.8)	18 (17.2)	0.419
Hyperlipidemia (n, %)	67 (40.6)	50 (74.6)	17 (25.4)	0.108
Coronary artery disease (n, %)	25 (15.2)	19 (76.0)	6 (24.0)	0.527
Pulmonary hypertension (n, %)	5 (3.0)	5 (100.0)	0 (0.0)	0.584
Atrial flutter/fibrillation (n, %)	17 (10.3)	16 (94.1)	1 (5.9)	0.199
Venous thromboembolism (n, %)	23 (13.9)	19 (82.6)	4 (17.4)	>0.999
Chronic kidney disease (n, %)	40 (24.2)	32 (80.0)	8 (20.0)	0.911
COPD (n, %)	35 (21.2)	29 (82.9)	6 (17.1)	0.813
ILD (n, %)	4 (2.4)	3 (75.0)	1 (25.0)	0.582
OSA/OHS (n, %)	37 (22.4)	31 (83.8)	6 (16.2)	0.645
CVA/TIA (n, %)	29 (17.6)	20 (69.0)	9 (31.0)	0.081
Malignancy (n, %)	27 (16.4)	22 (81.5)	5 (18.5)	>0.999
ARDS severity at 24 h				
Mild (n, %)	43 (26.1)	31 (72.1)	12 (27.9)	0.101
Moderate (n, %)	61 (37.0)	53 (86.9)	8 (13.1)	0.154
Severe (n, %)	14 (8.5)	12 (85.7)	2 (14.3)	>0.999
Days on supportive oxygen therapy (Mean, SD)	25.5 (19.3)	26.4 (18.1)	22.1 (23.1)	0.335
Days on NC (Mean, SD)	5.0 (6.7)	5.0 (6.8)	5.2 (6.5)	0.883
Days on HFNC (Mean, SD)	2.2 (3.4)	2.5 (3.6)	0.9 (1.6)	0.001*
Days on BIPAP (Mean, SD)	1.2 (3.8)	1.4 (4.3)	0.2 (0.5)	0.007*
Days on mechanical ventilation (Mean, SD)	17.8 (16.1)	18.4 (15.4)	15.9 (18.6)	0.502
Outcomes				
Tracheostomy (n, %)	44 (29.1)	38 (86.4)	6 (13.6)	0.190
Extubation (n, %)	71 (49.3)	58 (81.7)	13 (18.3)	0.734
Discharge home (n, %)	66 (40.0)	56 (84.9)	10 (15.2)	0.260
Discharge to LTAC (n, %)	20 (12.1)	15 (75.0)	5 (25.0)	0.496
Death (n, %)	63 (42.6)	46 (73.0)	17 (27.0)	0.173
Ventilator settings (24 h)				
RR (Mean, SD)	26.5 (6.3)	26.2 (6.2)	27.4 (6.8)	0.438
TV (Mean, SD)	388.3 (63.5)	393.1 (63.6)	369.4 (60.7)	0.087
FiO2 (Mean, SD)	0.52 (0.17)	0.52 (0.17)	0.49 (0.16)	0.270
PEEP (Mean, SD)	11.5 (3.8)	11.5 (3.5)	11.4 (4.6)	0.900
Peak airway pressure (Mean, SD)	28.5 (6.9)	28.4 (6.7)	28.8 (8.1)	0.807
Average airway pressure (Mean, SD)	16.6 (5.0)	16.5 (4.8)	16.6 (6.1)	0.933
Plateau pressure (Mean, SD)	24.2 (5.3)	24.0 (4.9)	24.9 (6.9)	0.558
Driving pressure (Mean, SD)	12.5 (4.2)	12.3 (3.8)	13.1 (5.6)	0.544
Ventilator settings (Peak)				
RR (Mean, SD)	31.5 (6.5)	31.7 (6.6)	31.1 (6.4)	0.685
TV (Mean, SD)	434.2 (86.6)	438.9 (87.8)	415.9 (80.4)	0.192
FiO2 (Mean, SD)	0.75 (0.26)	0.75 (0.26)	0.77 (0.25)	0.735
PEEP (Mean, SD)	13.3 (4.4)	13.5 (4.4)	12.2 (3.9)	0.117
MAP (Mean, SD)	22.0 (7.3)	22.0 (6.1)	22.1 (11.0)	0.971
Peak airway pressure (Mean, SD)	42.4 (11.8)	42.2 (11.7)	42.9 (12.3)	0.808
Plateau pressure (Mean, SD)	30.6 (7.6)	31.2 (7.4)	28.0 (8.0)	0.067
Driving pressure (Mean, SD)	17.2 (5.7)	17.6 (5.6)	15.7 (6.0)	0.147
Lowest P:F ratio	104.5 (56.7)	102.2 (55.2)	113.5 (62.3)	0.385
ICU complications				
Secondary pneumonia	79 (47.9)	63 (79.8)	17 (20.3)	0.789
Central line Ass. blood stream infection	14 (8.5)	11 (78.6)	3 (21.4)	0.736
Other infection	43 (26.1)	34 (79.1)	9 (20.9)	0.767
Pneumothorax	11 (6.7)	8 (72.7)	3 (27.3)	0.448
Supraventricular tachycardia	60 (36.4)	48 (80.0)	12 (20.0)	0.882
Cardiac ischemia acute kidney injury	39 (23.6)	28 (71.8)	11 (28.2)	0.111
With renal replacement therapy	45 (27.3)	39 (86.7)	6 (13.3)	0.228
Without renal replacement therapy	56 (33.9)	44 (78.6)	12 (21.4)	0.636
Acute liver disease	25 (15.1)	18 (72.0)	7 (28.0)	0.237
Venous thromboembolism	25 (15.2)	20 (80.0)	5 (20.0)	0.934
Delirium	52 (31.5)	36 (69.2)	16 (30.8)	0.012*
Hyperglycemia	86 (52.1)	76 (88.4)	10 (11.6)	0.009*
Gastrointestinal bleed	21 (12.7)	19 (90.5)	2 (9.5)	0.374
Other bleed	19 (11.5)	16 (84.2)	3 (15.8)	>0.999

The difference between peak ventilator settings and those at 24 h was examined between survivors and non-survivors. There was a greater change in mean airway pressures and PEEP in non-survivors compared to survivors (p=0.009 and 0.040 respectively; Table 5).

**Table 5:** Ventilator settings and mortality in patients with COVID-19 ARDS. COVID-19, coronavirus disease 2019; ARDS, acute respiratory distress syndrome *Indicates a statistically significant result at ⍺ = 0.05

Change in Ventilator Properties (Peak – 24 h)	Total Population (n = 165)	Survivors (n = 85)	Non-survivors (n = 63)	Significance (p-value)
Respiratory rate	4.90 (5.86)	4.56 (6.42)	5.20 (5.19)	0.541
Tidal volume	46.87 (66.50)	48.01 (61.48)	45.20 (72.92)	0.820
Positive end-expiratory pressure	1.74 (3.22)	1.21 (2.29)	2.47 (4.04)	0.040*
Increase in fractional inspired oxygen	0.24 (0.24)	0.22 (0.24)	0.26 (0.23)	0.330
Peak airway pressure	13.41 (11.37)	11.70 (10.26)	14.89 (11.17)	0.104
Mean airway pressure	5.40 (6.12)	4.06 (4.43)	7.00 (7.18)	0.009*
Plateau pressure	6.01 (6.48)	5.40 (4.40)	6.57 (8.41)	0.371
Driving pressure	4.31 (5.59)	4.26 (4.14)	4.10 (6.93)	0.886

The complications of COVID-19-associated ARDS patients are summarized in Table 6. Those who survived had a higher incidence of secondary pneumonia and delirium.

**Table 6:** Complications of patients with COVID-19 ARDS. COVID-19, coronavirus disease 2019; ARDS, acute respiratory distress syndrome *Indicates a statistically significant result at ⍺ = 0.05

Characteristic	Total Population (n = 165)	Survivors (n = 85)	Non-survivors (n = 63)	Significance (p-value)
Secondary pneumonia	79 (47.9)	52 (67.5)	25 (32.5)	0.010*
Central line associated blood stream infection	14 (8.5)	7 (58.3)	5 (41.7)	0.947
Other infection	43 (26.1)	24 (55.8)	19 (44.2)	0.799
Pneumothorax	11 (6.7)	6 (54.5)	4 (45.5)	0.841
Supraventricular tachycardia	60 (36.4)	31 (52.5)	28 (47.5)	0.327
Cardiac ischemia acute kidney injury	39 (23.6)	19 (51.4)	18 (48.7)	0.388
With renal replacement therapy	45 (27.3)	20 (45.5)	24 (54.5)	0.055
Without renal replacement therapy	56 (33.9)	29 (51.8)	27 (48.2)	0.278
Acute liver disease	25 (15.1)	12 (48.0)	13 (52.0)	0.295
Venous thromboembolism	25 (15.2)	17 (73.9)	6 (26.1)	0.082
Delirium	52 (31.5)	36 (70.6)	15 (29.4)	0.019*
Hyperglycemia	86 (52.1)	51 (59.3)	35 (40.7)	0.588
Gastrointestinal bleed	21 (12.7)	13 (61.9)	8 (38.1)	0.655
Other bleed	19 (11.5)	10 (55.6)	8 (44.4)	0.864

Multiple correlations were analyzed between hospital length of stay and modality of oxygenation/ventilation using Pearson’s r statistic. Increasing ICU days were correlated with increasing hospital days overall (r=0.904, p<0.001). Increased days on mechanical ventilation were associated with overall increased hospital stay (r=0.813, p<0.001). Increased days on HFNC and BIPAP were associated with fewer ICU days (r=0.183, p=0.036 and p=0.268, p=0.003 respectively). Increased days on BIPAP were also associated with overall lower hospital length of stay (r=0.232, p=0.011).

## Discussion

In this retrospective study, we have attempted to explore the clinical characteristics and outcomes of ARDS patients in an urban population during the early phase of the COVID-19 pandemic. In terms of our demographics, our study had an average age of 64 for the total cohort, which is consistent with prior studies that have also reported an average age of intubated patients of around 64-68 years of age [5-6, 8]. Also consistent with prior studies -- hypertension, type 2 diabetes mellitus, and obesity were the most common co-morbidities [8]. We found that having chronic obstructive pulmonary disease (COPD) as a comorbidity was associated with higher mortality. The COPD patients have demonstrated to have nearly twice the severity of COVID-19 infection and higher mortality in three recent meta-analysis [9-11]. This highlights the importance of preventative management and targeted vaccination strategies towards patients with COPD. We found an in-hospital mortality of 38.2%, which is consistent with similar studies in the early phase of the pandemic that have suggested mortality rates of 27.2%-61.5% [5, 8,12-13]. The wide range of mortality may be due to the different endpoints used in studies that are typically either in-hospital or 30-day mortality. It was unclear how much lack of resources (i.e., ventilators) contributed to higher mortality due to increased demand from pandemic-related hospital surges.

Higher FiO2 requirements at 24 h were associated with an increase in in-hospital mortality as reflected by 0.48 in survivors and 0.56 in non-survivors (p=0.003). Ferrando et al. (2020) also demonstrated that patients with higher FiO2 requirements on the first day of invasive mechanical ventilation correlated with ARDS severity that correlated with a higher 28-day mortality [8]. Higher plateau pressures, 23.2 cmH2O in survivors vs. 25.6 cmH2O in non-survivors (p= 0.023), at 24 h from intubation was associated with higher in-hospital mortality. Ferrando et al. (2020) also demonstrated that patients with higher maximum plateau pressures on the first day of invasive mechanical ventilation correlated with ARDS severity, which correlated with a higher 28-day mortality [8]. Higher driving pressures at 24 h were also found to correlate with in-hospital mortality in our study, 11.8 cmH2O in survivors vs. 13.5 cmH2O in non-survivors (p=0.048). This was not found to be statistically significant in the Ferrando et al. (2020) study. Likely differences in findings are related to different measured endpoints as our study primarily looked at in-hospital mortality and their study stratified based on ARDS severity.

Higher mean airway pressures, peak airway pressure, and plateau pressures after 24 h also correlated with higher in-hospital mortality. Not surprisingly, lower P:F ratios also correlated with increased in-hospital mortality. The significance of these findings on ventilator settings highlights the importance of low stretch ventilation. The aim is to keep the lowest possible intrathoracic pressures to minimalize ventilator-induced lung injury during the reduced pulmonary compliance experienced during ARDS. When examining the change in peak ventilator settings from 24 h settings, we saw no significant change in driving pressures between survivors and non-survivors, however, there were significantly higher changes in both PEEP and mean airway pressure. This possibly represents a decline in airway compliance leading to mortality.

Early studies from Wuhan, China initially cautioned the use of systemic corticosteroids due to reported increased rate of myocardial and liver injury, shock, need for mechanical ventilation, and higher 28-day all-cause mortality [1]. Thus, the initiation of corticosteroids was highly controversial at the onset of the pandemic. Despite the controversy, one of the earliest therapeutics utilized for critically ill patients was corticosteroids, with several studies demonstrating mortality benefits [14-16]. A study from Brazil evaluated the efficacy of methylprednisolone to placebo among hospitalized patients with COVID-19, and the reported 28-day mortality was no different between groups [17]. Another study from Iran compared the outcomes of methylprednisolone to dexamethasone and reported a statistically significant improved clinical status in those who received methylprednisolone compared to those who received dexamethasone [18]. A meta-analysis from JAMA included several retrospective studies intending to elucidate the impact of different systemic corticosteroids on critically ill patients with COVID-19 [15]. Each individual study enrolled patients in either hydrocortisone, methylprednisolone, or dexamethasone. This meta-analysis reported dexamethasone as the most effective corticosteroid for reducing mortality in these patients, with an odds ratio (OR) of 0.64, compared to 0.69 and 0.91 for hydrocortisone and methylprednisolone respectively [15]. However, this study imprecisely accounted for variability in dose and treatment duration. Moreover, there was no evidence suggesting that higher doses were associated with greater benefits than lower-dose steroids. There is great variability in patient enrolment, treatment selection, and contrasting outcomes in the literature. 

Limitations

Our study has several limitations that could have influenced its findings. It is limited by retrospective design, small power, and restriction to one health system. Due to the evolving nature of the pandemic and the reliance on EMR, it is possible that coding errors could have missed eligible patients within our health system. The population our healthcare system serves is uniquely suited to those of the underserved in the state of Pennsylvania. That being said, a majority of our population identified as black, which possibly affects the generalizability of the data. In addition, a very small proportion of overall patients were afflicted with either pulmonary hypertension or interstitial lung disease, and our data may not specifically apply to ventilator management and ARDS in these patients, who both have altered lung and cardiovascular physiology. Our study is also limited by its applicability since its data was collected from early in the pandemic. Treatments have evolved over the pandemic and vaccinations have become readily available after this data was collected. Hence, our findings may not be fully reflective of current COVID-19-associated ARDS patients.

## Conclusions

COVID-19-associated ARDS is associated with significant mortality. Physicians should be aware of pre-existing conditions that are potentially associated with worse outcomes so that they receive the appropriate level of care in a timely manner. Ventilator management should focus on previous ARDS strategies to reduced ventilator induced lung injury, including striving for lower plateau pressures and driving pressures. More observational studies are needed to guide COVID-19-associated ARDS management.

## Appendices

**Table 7:** Clinical variables. ARDS, acute respiratory distress syndrome; COVID-19, coronavirus disease 2019; COPD, chronic obstructive pulmonary disease; ILD, interstitial lung disease; TIA, transient ischemic attack; P:F, pressure of arterial oxygen to fraction of inspired oxygen

Body mass index	Calculated as kg/m^2^ using the patient’s recorded body weight and height on admission
Comorbid conditions	Common comorbid conditions were selected for analysis, including the following: hypertension, diabetes mellitus, hyperlipidemia, coronary artery disease, pulmonary hypertension, atrial fibrillation/flutter, venous thromboembolism, chronic kidney disease, COPD, ILD, other pulmonary disorder, obstructive sleep apnea, obesity hypoventilation syndrome, cerebral vascular accident/TIA, and malignancy.
Steroid therapy	High-dose steroid therapy was defined as hydrocortisone 50 mg every 6 h or 100 mg every 8 h or more, methylprednisone at least 0.5 mg/kg per day, dexamethasone 1-2 mg/kg initially or 10-20 mg daily, with minimum duration of 3 days.
Supportive oxygen therapy	Defined as days requiring supplemental oxygen in the form of nasal cannula, high-flow via nasal cannula, non-invasive positive pressure ventilation, or invasive mechanical ventilation. Data taken from electronic respiratory flowsheets.
Ventilatory settings at 24 h	Recorded ventilatory settings 24 h after intubation of patient. Data taken from electronic respiratory flowsheets.
Peak ventilatory settings	Highest recorded ventilatory settings during the course of intubation. Data taken from electronic respiratory flowsheets.
ARDS severity	Determined using the Berlin criteria for ARDS as mild, moderate, and severe using the patient’s initial PaO2/FiO2 ratio on a positive end expiratory pressure of 5 cmH2O or high flow nasal cannula. A P:F ratio <300, <200 or <100 was defined as mild, moderate, and severe, respectively.
Complications of patients with COVID-19 ARDS	Common complications of critically ill patients were assessed, including secondary pneumonia, central line associated blood stream infection, other infection, pneumothorax, supraventricular tachycardia, cardiac ischemia, acute kidney injury (with and without renal replacement therapy), acute liver disease, venous thromboembolism, delirium, hyperglycemia, gastrointestinal bleed, or other bleed.
